# Changes in Tobacco Use, Susceptibility to Future Smoking, and Quit Attempts among Canadian Youth over Time: A Comparison of Off-Reserve Aboriginal and Non-Aboriginal Youth

**DOI:** 10.3390/ijerph10020729

**Published:** 2013-02-21

**Authors:** Tara Elton-Marshall, Scott T. Leatherdale, Robin Burkhalter, K. Stephen Brown

**Affiliations:** 1 School of Public Health and Health Systems, University of Waterloo, Waterloo, ON N2L 3G1, Canada; E-Mails: teelton@uwaterloo.ca (T.E.-M.); ksbrown@uwaterloo.ca (K.S.B.); 2 Propel Centre for Population Health Impact, University of Waterloo, Waterloo, ON N2L 3G1, Canada; E-Mail: rjburkhalt@uwaterloo.ca; 3 Department of Statistics and Actuarial Sciences, University of Waterloo, Waterloo, ON N2L 3G1, Canada

**Keywords:** aboriginal, adolescent, youth, smoking, quit attempts, smoking susceptibility

## Abstract

The purpose of this study was to determine whether there is a growing inequity in tobacco use, susceptibility to future smoking, and quit attempts among Off-Reserve Aboriginal (ORA) youth in Canada relative to Non-Aboriginal youth. Current smoking, susceptibility to future smoking and quit attempts were examined among a nationally representative sample of ORA and Non-Aboriginal Canadian youth. Data are from cross-sectional surveys of 88,661 respondents in Grades 6 to 9 across the 2004, 2006 and 2008 survey waves of the Youth Smoking Survey (YSS). At each wave, ORA youth were more likely to be current smokers (overall OR = 3.91, 95% CI 3.47 to 4.41), to be susceptible to future smoking (overall OR = 1.37, 95% CI 1.27 to 1.48), and less likely to have ever made a quit attempt compared to Non-Aboriginal youth (overall OR = 0.74, 95% CI 0.57 to 0.96). Although susceptibility to future smoking declined for Non-Aboriginal youth, the prevalence of susceptibility remained stable among ORA youth. The percentage of ORA youth reporting making a quit attempt increased, however, current smoking rates among ORA youth did not decline. These findings suggest that the disparity in susceptibility to future tobacco use among ORA and Non-Aboriginal youth has increased over time. Despite increased rates of quit attempts, current smoking rates remain significantly higher among ORA youth. Tobacco control programs for Aboriginal youth should be a public health priority.

## 1. Introduction

Tobacco use is the single most preventable cause of death [1,2]. Within Canada, the risk of dying from tobacco-related diseases is significantly higher for Aboriginal Canadians compared to the rest of the population [3,4,5]. Aboriginal Canadian youth living on reserves have significantly higher rates of tobacco use compared to Non-Aboriginal youth [6,7,8] and are more likely to initiate smoking at an earlier age [7]. This is cause for concern as earlier smoking initiation is associated with an increased risk of developing chronic diseases over time [9,10,11].

A recent review of the research literature on Aboriginal health revealed a significant under-representation of research among First Nations living off-reserve and a lack of research focused on the unique health needs of Aboriginal women and children [12]. Tobacco control research among Off-Reserve Aboriginals (ORA) is particularly important because the majority of Aboriginal Canadians live off-reserve [13] and few studies have examined smoking among ORA youth in Canada [14,15,16]. Most recently, data from the Youth Smoking Survey (YSS) identified that the prevalence of current smoking among ORA youth was nearly double that among Non-Aboriginal youth (24.9% *vs.* 10.4%) and that ORA youth were less likely to have ever made a quit attempt [17]. This research demonstrates the importance of developing appropriate tobacco control policies and programs for Aboriginal youth attending schools off-reserves. However, a limitation of this research is that it is unknown whether tobacco use among ORA youth has declined over time or whether disparities between ORA and Non-Aboriginal youth smoking have actually improved. This study will therefore examine smoking behaviour among a nationally representative sample of youth over time (2004, 2006, and 2008) using data from the Youth Smoking Survey. The purpose of this study was to determine whether there is a growing inequity in tobacco use, susceptibility to future smoking, and quit attempts among Off-Reserve Aboriginal (ORA) youth in Canada relative to Non-Aboriginal youth.

## 2. Methods

### 2.1. Study Design

Data were collected from 88,661 respondents in Grades 6 to 9 across the 2004 (n = 23,362), 2006 (n = 34,050), and 2008 (n = 31,249) survey waves as part of the Youth Smoking Survey (YSS) [18]. Grades 6 to 9 were included because these were the grades that were consistently measured across all survey years. The target population was youth attending public and private schools in the 10 Canadian provinces. Schools in the Yukon, Nunavut and the Northwest Territories, were excluded as were youth living in institutions or on First Nation Reserves, and youth attending special schools or schools on military bases. Given that this is a secondary data analysis and inclusion of these individuals was not part of the primary aim of the Youth Smoking Survey, we were unable to examine smoking among Aboriginal youth in these regions.

In general, the sampling design for each wave was stratified based on the health region (or in 2004, school board) smoking rate. The Canadian Community Health Survey (CCHS) was used to calculate the smoking rate among 15 to 19 year olds for each health region. The school lists obtained from the provincial Departments of Education for each of the 10 provinces included enrolment data by grade for each school. Using this list, the total eligible grade enrolment in a health region was used as a weight to compute the median smoking rate for each province. Each school’s six-digit postal code was used to identify the health region in which it was located. Schools were then categorized as “low” or “high” smoking rate stratum based on the smoking rate in their health region compared to the median (where greater than or equal to the median was categorized as “high”). Within each participating school, all students in the survey grades were eligible to participate. Research ethics approval for this study was obtained from the University of Waterloo Office of Research Ethics and local school boards as required. Detailed information about the sample design and survey methods across survey waves are available elsewhere [18].

### 2.2. Measures

Consistent with previous research [17], Off-Reserve Aboriginal status was determined by asking respondents: Are you an Aboriginal person? (Yes, First Nations; Yes, Métis; Yes, Inuit; No, I am not an Aboriginal person). Respondents also reported their current grade, sex, smoking status and weekly spending money. Smoking status was based on Health Canada’s definitions of smoking status [19]. Current smokers had smoked at least 100 cigarettes in his or her lifetime and had smoked at least one whole cigarette during the past 30 days. Non-smokers had not smoked 100 or more whole cigarettes in his or her lifetime but might have smoked a whole cigarette. 

Susceptibility to future smoking among those who had never tried smoking was measured using the validated algorithm of Pierce and colleagues [20]. Susceptibility was measured by asking students: “Do you think in the future you might try smoking cigarettes?”, “If one of your best friends were to offer you a cigarette, would you smoke it?”, and “At any time during the next year do you think you will smoke a cigarette?” Students responded to these questions on a 4-point Likert scale. Consistent with Pierce and colleagues [20] students who answered ‘definitely not’ to all three questions were considered non-susceptible; they were considered susceptible to future smoking if they responded positively to at least one item. Current smokers also reported whether they had ever tried to quit (0 = I have never tried to quit, 1 = I have tried to quit 1 or more times). These tobacco use items have been used in other youth smoking surveys (e.g., Global Youth Tobacco Survey, Ontario Student Drug Use and Health Survey) and have been established as reliable and valid. Additional information on the YSS measures is available online (www.yss.uwaterloo.ca).

### 2.3. Survey Protocol

Teachers administered the pen and paper survey using standardized protocols during a designated class period. To ensure confidentiality and therefore encourage honest responses, teachers were asked to avoid circulating among the students. Students were also required to place their completed survey in an envelope and seal this envelope before it was collected by a student in the classroom. Prior to implementation, the survey questionnaire was pilot tested (in both French and English). During the two-hour pilot testing sessions, students representing smokers and non-smokers from all grades completed the questionnaire independently and were encouraged to write comments/questions while doing so. Respondents then participated in a 75 min focus group discussion in their first language led by a moderator using a pre-developed survey guide. The moderator explored students’ comprehension of the survey questions (with particular focus on all new questions), the logic and order of the questions, and overall flow of the questionnaire. 

### 2.4. Analyses

Weighted chi-square analyses were conducted to test for differences between our samples over the 2004, 2006, and 2008 survey waves and between Aboriginals and Non-Aboriginals in prevalence rates of: current smoking, susceptibility to future smoking, and ever making a quit attempt. Former smokers (n = 381) were excluded from definitions of smoking status and eliminated from analyses due to sample sizes too small to include in our statistical models. Generalized Linear Mixed Models using PROC GLIMMIX in SAS were used to test whether Aboriginal status predicted key outcome variables after controlling for demographic variables (sex, grade, region, weekly spending money, survey year) and adjusting for clustering within schools. Survey weights were used to adjust for non-response between provinces and groups, (thereby minimizing any bias in the analyses caused by differential response rates across regions or groups) and to provide population-level prevalence estimates. The statistical package SAS 9.2 was used for all analyses.

## 3. Results

### 3.1. Demographics

A total of 6,901 respondents in the sample self-identified as Aboriginal (4.2% of the sample in 2004, 8.1% in 2006 and 7.3% in 2008). Table 1 presents the weighted sample characteristics of respondents in our sample by year and Aboriginal status.

### 3.2. Prevalence of Smoking Behaviour among ORA and Non-Aboriginal Youth Over Time

**Figure 1:**
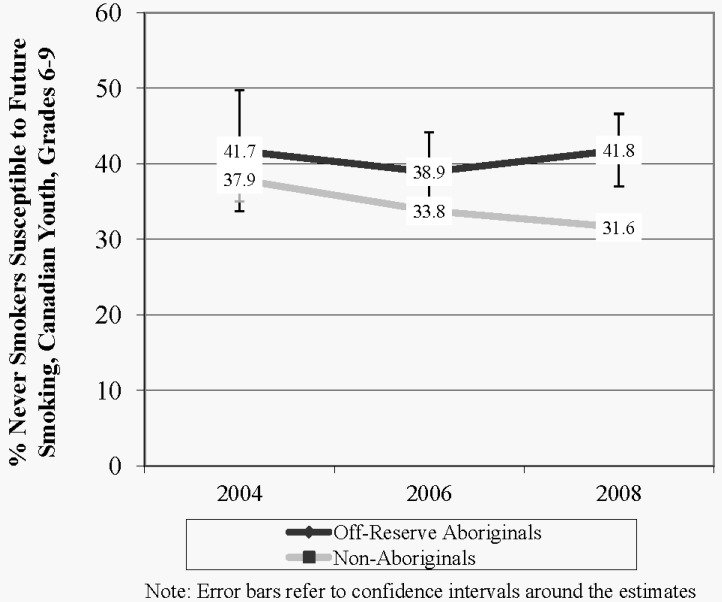
Susceptibility to future smoking among never smoking Aboriginal and Non-Aboriginal youth in grades 6 to 9, Canada, 2004–2008.

As shown in Figure 1, susceptibility to future smoking among never smokers decreased between 2004 and 2008 among Non-Aboriginal youth (by 16.7%) whereas susceptibility remained high among ORA youth (0.2% increase). Overall, susceptibility to future smoking was consistently higher among ORA compared to Non-Aboriginal youth within each survey wave. 

**Table 1:** Descriptive statistics for the sample of youth (grades 6 to 9) Aboriginal and Non-Aboriginal Youth, 2004-2008, Canada.

	2004 (n = 23,362)			2006 (n = 34,050)			2008 (n = 31,249)		
	**Aboriginal** **^a^**	**Non-Aboriginal** **^a^**	**Chi-Square**	**Aboriginal** **^a^**	**Non-Aboriginal** **^a^**	**Chi-Square**	**Aboriginal** **^a^**	**Non-Aboriginal** **^a^**	**Chi-Square**
**Sex**									
Male	54.2	50.9	Х^2^ = 4.00 (1), p = 0.05	52.9	50.9	Х^2^ = 4.00 (1), p = 0.05	52.4	50.7	Х^2^ = 2.51 (1), p = 0.11
Female	45.8	49.1		47.1	49.1		47.6	49.3	
**Grade**									
6	24.7	24.0	Х^2^ = 22.9 (3), p < 0.001	23.4	21.8	Х^2^ = 8.92 (3), p = 0.03	23.1	22.9	Х^2^ = 5.49 (3), p = 0.14
7	19.8	25.6		24.8	24.7		24.8	24.7	
8	24.9	25.2		23.9	26.5		23.9	25.9	
9	30.7	25.2		27.9	27.0		28.2	26.5	
**Weekly**									
**spending**									
**money**									
$0	23.9	21.9	Х^2^ = 42.9 (3), p < 0.001	13.4	19.1	Х^2^ = 104.4 (3), p < 0.001	17.5	23.2	Х^2^ = 146.75 (3), p < 0.001
$1 to $20	37.4	46.9		50.2	49.0		40.4	44.4	
$21 or more	26.7	19.3		27.6	20.8		29.3	18.9	
Don’t know	12.0	11.9		8.8	11.1		12.8	13.4	

^a^ weighted population estimate

**Figure 2:**
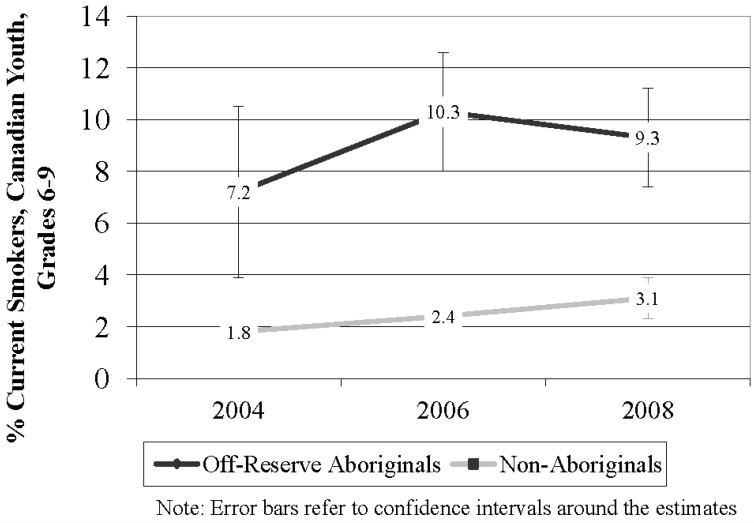
Smoking prevalence among Aboriginal and Non-Aboriginal youth in grades 6 to 9, Canada, 2004–2008.

As shown in Figure 2, current smoking prevalence was substantially higher among ORA youth in every survey wave although rates of current smoking increased between 2004 and 2008 among both ORA (29.2% increase) and Non-Aboriginal youth (72.2% increase).

**Figure 3:**
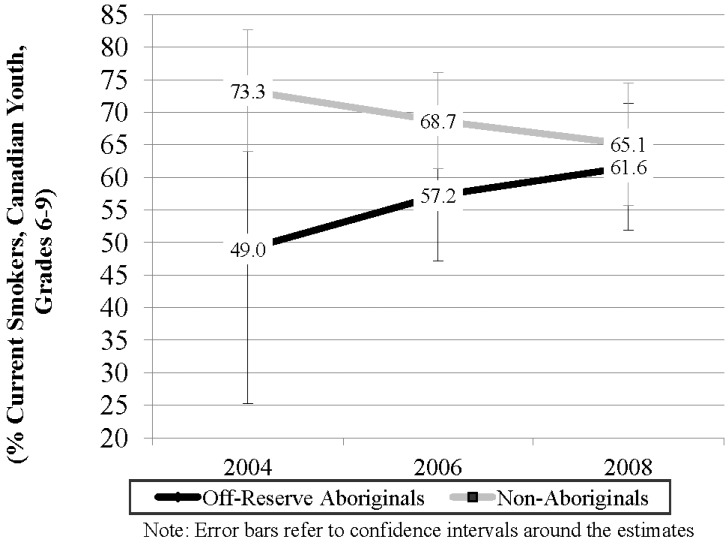
Ever made a quit attempt among Aboriginal and Non-Aboriginal current smokers in grades 6 to 9, Canada 2004–2008.

As shown in Figure 3, ORA youth had lower rates of having ever made a quit attempt within each survey wave. However, rates of having ever made a quit attempt increased between 2004 and 2008 among ORA youth (25.7%) and decreased among Non-Aboriginal youth (11.1%). 

### 3.3. Testing the Differences in Smoking Behaviour among ORA and Non-Aboriginal Youth

We tested whether there were differences in smoking behaviour among all youth over time and by Aboriginal status (Table 2). Model 1 predicted the likelihood of being a current smoker *versus* a non-smoker. Compared to a respondent in 2004, a respondent in 2008 was significantly more likely to be a current smoker (p < 0.01, OR 1.55, 95% CI 1.20 to 1.99). ORA youth were also significantly more likely to be current smokers compared to Non-Aboriginal youth (p < 0.001, OR = 3.91 95% CI 3.47 to 4.41). 

Model 2 predicted the likelihood of a never smoker being susceptible to future smoking *versus* not being susceptible to future smoking. Compared to a never smoker in 2004, never smokers in 2006 (p < 0.01, OR 0.78, 95% CI 0.71 to 0.86) and 2008 (p < 0.01, OR 0.79, 95% CI 0.72 to 0.87) were less likely to be susceptible to smoking. Never smoking ORA youth were more likely to be susceptible to future smoking compared to Non-Aboriginal youth (p < 0.001, OR = 1.37, 95% CI 1.27 to 1.48). 

Model 3 predicted the likelihood of ever making a quit attempt *versus* never making a quit attempt among current smokers. There was no significant difference in the likelihood of ever making a quit attempt over time. ORA youth were less likely to have ever made a quit attempt compared to Non-Aboriginal youth (p = 0.02, OR = 0.74, 95% CI 0.57 to 0.96).

**Table 2:** Weighted generalized linear mixed model analyses examining current smoking and ever making a quit attempt among Aboriginal and Non-Aboriginal youth in grades 6–9, 2004–2008, Canada.

		Adjusted odds ratio* (95% CI)		
		Model 1	Model 2	Model 3
Parameters		Current smoking *vs.* Non-smoking	Susceptibility to future smoking *vs.* Non-Susceptible ^†^	Ever made a quit attempt *vs.* Never made a quit attempt ^‡^
**Sex**				
	Male	1.00	1.00	1.00
****	Female ****	0.87 (0.80 to 0.95) ^||^	0.97 (0.94 to 1.01)	1.91 (1.56 to 2.33) ^¶^
**Grade**				
	6	1.00	1.00	1.00
	7	5.30 (3.64 to 7.71) ^¶^	1.21 (1.15 to 1.27) ^¶^	1.47 (0.58 to 3.76)
	8	12.77 (8.86 to 18.41) ^¶^	1.26 (1.19 to 1.33) ^¶^	1.35 (0.54 to 3.35)
****	9	22.77 (15.80 to 32.83) ^¶^	1.09 (1.02 to 1.17) ^§^	1.22 (0.49 to 3.03)
**Weekly Spending Money**				
	$0	1.00	1.00	1.00
	$1 to $20	1.78 (1.52 to 2.09) ^¶^	1.12 (1.08 to 1.17) ^¶^	1.65 (1.15 to 2.37) ^||^
	$21 or more	3.80 (3.25 to 4.44) ^¶^	1.43 (1.35 to 1.51) ^¶^	1.07 (0.75 to 1.52)
****	Don’t know ****	2.05 (1.70 to 2.48) ^¶^	1.08 (1.02 to 1.15) ^§^	1.63 (1.04 to 2.54) ^§^
**Survey Year**				
	2004	1.00	1.00	1.00
	2006	1.24 (0.97 to 1.59)	0.78 (0.72 to 0.86) ^¶^	0.95 (0.58 to 1.55)
****	2008	1.54 (1.20 to 1.99) ^¶^	0.79 (0.72 to 0.87) ^¶^	0.91 (0.55 to 1.49)
**Aboriginal status**				
	Non-Aboriginal	1.00	1.00	1.00
	Aboriginal	3.91 (3.47 to 4.41) ^¶^	1.37 (1.27 to 1.48) ^¶^	0.74 (0.57 to 0.96) ^§^

Model 3: 1 = Ever made a quit attempt (n = 1,957), 0 = Never made a quit attempt (n = 844). Model 2: 1 = Susceptible to future smoking (n = 21,887), 0 = Not susceptible to future smoking (n = 46,392) * Odds ratios adjusted for all other variables in the table and controlling for region † Among respondents who have never tried; ‡ Among current smokers; ^§^ p < 0.05, ^||^ p < 0.01, ^¶^ p < 0.001; Model 1: 1 = Current smoker (n = 3,026), 0 = Non-Smoker (n = 85,254).

## 4. Discussion

This was the first study to examine tobacco use over time among ORA youth in Canada. Consistent with previous research among an older sample of youth [17], ORA youth in Grades 6 to 9 have higher prevalence rates of current smoking and lower prevalence rates of having ever made a quit attempt compared to Non-Aboriginal youth in Canada. A higher prevalence of smoking among Aboriginal youth relative to Non-Aboriginal youth was found in other countries such as the United States where 16.8% of American Indian or Alaska Native youth ages 12-17 use cigarettes compared to the national average of 10.2% [21]. Similarly, Indigenous adults in Australia are more than twice as likely to be current smokers compared to Non-Indigenous adults [22]. 

Previous research among Aboriginal Australian adults found that smoking rates decreased slightly (from 53.1% to 49.8%) between 2002 and 2008 and the proportion of ex-smokers increased (from 16.5% to 21.4%) during this time [22]. Cigarettes smoked per day also decreased for youth ages 15–24 from 1994–2008 for those in the heaviest smoking category [23]. The current study did not find decreases in smoking prevalence among Aboriginal youth in Canada over time. Additionally, Indigenous and non-Indigenous populations were not compared in the Australian studies. Therefore it is unknown whether changes over time were the result of a cohort effect and whether the disparities in smoking status remain. In contrast, the current study was able to demonstrate a growing disparity among ORA and Non-Aboriginal youth for those most at risk for future smoking. An international examination of the problem of smoking among Indigenous populations may elucidate common reasons for these disparities and potential solutions.

Susceptibility to future smoking is a critical measure of prevention because it provides an indication of how likely non-smoking youth are to become future smokers [20]. Although susceptibility to future smoking among non-smoking youth declined for Non-Aboriginal youth between 2004 and 2008, the prevalence of susceptibility actually remained relatively stable among the population of ORA youth. Given that susceptibility to future smoking is predictive of future smoking initiation, these findings suggest that the gap in smoking prevalence rates could widen as many of the susceptible ORA youth start smoking. Prevention initiatives targeting ORA youth are also urgently needed to reduce the likelihood of ORA youth progressing to smoking experimentation. 

We identified that although ORA current smokers were less likely to have ever made a quit attempt relative to Non-Aboriginal youth, the percentage of ORA youth reporting making a quit attempt increased dramatically from 2004 to 2008. This finding is consistent with previous research demonstrating that the majority of ORA youth in British Columbia are interested in quitting [14]. Although it is encouraging that more ORA youth are making quit attempts, there have been no significant changes in smoking rates among ORA over time. Current smoking rates increased among all youth between 2004 and 2008 with the odds of being a current smoker significantly higher for ORA youth compared to Non-Aboriginal youth. These findings suggest that ORA youth may not be successful in their quit attempts.

Previous research has suggested that factors such as: colonization, poverty and economic marginalization, poor living conditions, lack of control, limited connectedness to mainstream society, and threats to traditional culture could increase the risk of substance use among the Aboriginal community [24,25]. Additionally, the increased prevalence of tobacco use among Aboriginal youth could lead to a normalization of smoking in the community and therefore encourage further use [24]. One potential strategy to improve tobacco control efforts is to develop more culturally-tailored tobacco use prevention and cessation approaches within the Aboriginal community [26]. School-level policies and programs that incorporate culturally appropriate messages could provide an opportunity to engage Aboriginal youth attending schools off-reserve. However, to date, there is a lack of evidence demonstrating the efficacy of tobacco prevention initiatives tailored for Aboriginal youth and more research is needed [27]. Given that the high prevalence of smoking in the Aboriginal community may lead to greater normalisation of smoking, community-wide interventions may be helpful although such strategies have not been evaluated within Aboriginal communities thus far [28]. 

### Limitations

This study uses a repeated cross-sectional design rather than a longitudinal cohort study and therefore represents changes in trends over time rather than changes within the same individuals. Data were also limited to youth in grades 6–9 and therefore cannot be generalized to students in higher or lower grades.

## 5. Conclusions

Substantially more effort is required to reduce the existing inequities between Aboriginal and Non-Aboriginal youth. Considering that self-identified Aboriginal people are the fastest growing population in Canada [29], and that Aboriginals are more likely to die from smoking-related diseases, these findings suggest that a rapid public health response is required to prevent further disparities in health burden. Given that the majority of Aboriginal youth have tried to quit smoking but that current smoking rates remain high, policies and programs to support cessation among Aboriginal youth are necessary. Previous research has demonstrated that cessation advice from physicians and other health care providers increases the likelihood that an individual will quit smoking [30,31]. Health care providers therefore need to screen for smoking status and be aware that Aboriginal youth are interested in quitting. Recently the Canadian Federal government cancelled funding for the National Aboriginal Health Organization which oversaw tobacco control initiatives sponsored by Health Canada. The current paper suggests that there is an urgent need for further programs targeting Aboriginal youth and that continued funding for tobacco control initiatives in the Aboriginal community are critical. 
